# The Use and Value of Muriate of Pilocarpin in Diphtheria

**Published:** 1886-11

**Authors:** 


### *THE USE AND VALUE OF MURIATE OF PILOCARPIN IN DIPHTHERIA.*

Dr. W. K. Harris, in the *Kansas City Medical Index*, details his experience with muriate of pilocarpin in the treatment of Diphtheria, having been many times successful with the formula of Guttman of Cronstadt, which is as follows:

| | | |
|---|---|---|
| ℞ Pilocarpin. Muriatis, - - | ⅓ to ⅔ grs. | |
| Acid Hydrochloric, - - - | gtt ij to v. | |
| Pepsin, - - - - | grs. i to iss. | |
| Aquæ Distillat, - - - | ℥ ijss. | M. |

Sig:—Teaspoonful to a child two years old, every hour.

While the pilocarpin is the principle remedy, there are useful adjuvants, of which calomel, from its well-known property of preventing inflammation and plastic exudation, holds first rank. This, with a solution of potassa chlorate as a gargle, is often enough to control the fetor.

If the patient be too young to use gargle, have the attendant give from one half to one teaspoonful by the mouth every hour or two.

The calomel should be given in from three to six-grain doses, every two to three hours, discontinuing it when the dark green discharges show the full effect of the mercury in the bowels.

*Apropos of Mercury.*—By directing the attendants to withhold all acid drinks, and have the patient click or snap his teeth together, and stop the exhibition of the calomel when he complains of a feeling of soreness or tenderness in the teeth, you need have no fear of creating ptyalism.

Dr. Guttman, in his report, says: "The action of pilocarpin is purely local, preventing the development of the membrane and organisms on which the disease is presumed to depend, dissolving and carrying them away by the excessive flow of saliva and mucous secretion."

We cannot coincide with the views of Dr. Guttman as to its being a purely local remedy. First, the diaphoresis which ensues almost immediately. Second, the decline in temperature and volume of pulse; still later, decrease in number of pulse-beats to the minute; and, if given in too large doses or pushed too closely, causing nausea and vomiting (simulating the effects of veratrum viride in large doses.) Sweating follows its exhibition in from fifteen to thirty minutes; the increased expectoration in an hour or so; and the fall in temperature, as shown by the thermometer, in from six to twelve hours, not to rise again during the course of the disease.

Its use is followed by excessive secretion from the mucous membrane of the throat and fauces melting and dissolving the patches of diphtheritic membrane which disappear and are carried away by the profuse flow of saliva in from thirty-six to forty-eight hours. Some small lingering patches may be discovered after that time. In only one case did the membrane continue longer and in only one case was it reproduced after once disappearing.

